# Valerian essential oil for treating insomnia *via* the serotonergic synapse pathway

**DOI:** 10.3389/fnut.2022.927434

**Published:** 2022-07-28

**Authors:** Wenfei Wang, Yichun Wang, Qiuting Guo, Huiting Li, Zhaoqiang Wang, Jia Li, Taotao Li, Tiantian Tang, Yujiao Wang, Yanzhuo Jia, Yao Wang, Junbo Zou, Yajun Shi, Dongyan Guo, Ming Yang, Xiaofei Zhang, Jing Sun

**Affiliations:** ^1^Key Laboratory of Basic and New Drug Research of Traditional Chinese Medicine, College of Pharmacy, Shaanxi University of Chinese Medicine, Xianyang, China; ^2^School of Pharmaceutical Sciences, College of Pharmacy, Kyushu University, Fukuoka, Japan; ^3^Xianyang Vocational Technical College, College of Pharmacy, Xianyang, China; ^4^Key Laboratory of Modern Preparation of TCM, Ministry of Education, College of Pharmacy, Jiangxi University of Chinese Medicine, Nanchang, China; ^5^Shaanxi Haitian Pharmaceutical Co., Ltd., Xianyang, China

**Keywords:** insomnia, valerian, transcriptome sequencing, weight coefficient, mechanism

## Abstract

Valerian volatile oil can be used in the treatment of insomnia; however, the active components and mechanisms of action are currently unclear. Therefore, we used transcriptome sequencing and weight coefficient network pharmacology to predict the effective components and mechanism of action of valerian volatile oil in an insomnia model induced by intraperitoneal injection of para-Chlorophenylalanine (PCPA) in SD rats. Valerian essential oil was given orally for treatment and the contents of 5-hydroxytryptamine receptor 1 A (5-HT1AR), γ-aminobutyric acid (GABA), cyclic adenosine monophosphate (cAMP), and protein kinase A (PKA) in the hippocampus of rats in each group were detected by enzyme-linked immunosorbent assay (ELISA), western blot, Polymerase Chain Reaction (PCR), and immunohistochemistry. The results showed that after treatment with valerian essential oil, insomnia rats showed significantly prolonged sleep duration and alleviated insomnia-induced tension and anxiety. Regarding the mechanism of action, we believe that caryophyllene in valerian essential oil upregulates the 5-HT1AR receptor to improve the activity or affinity of the central transmitter 5-HT, increase the release of 5-HT, couple 5-HT with a G protein coupled receptor, convert adenosine triphosphate (ATP) into cAMP (catalyzed by ADCY5), and then directly regulate the downstream pathway. Following pathway activation, we propose that the core gene protein kinase PKA activates the serotonergic synapse signal pathway to increase the expression of 5-HT and GABA, thus improving insomnia symptoms and alleviating anxiety. This study provides a theoretical basis for the application of valerian volatile oil in health food.

## Graphical Abstract

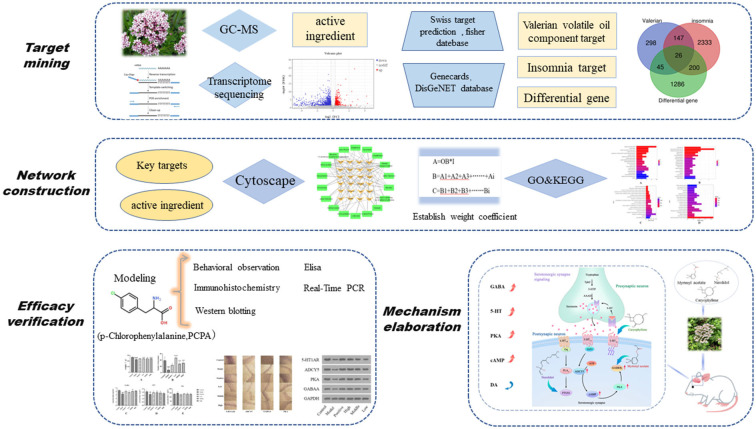

## Introduction

Insomnia, which can be caused by many factors, often related to physical diseases, is a common problem in today's society. Some studies also show that insomnia does great harm to our body and mind. Long-term insomnia will cause obstacles to human intelligence activities, such as memory loss, forgetfulness, inattention, and mental anxiety, all of which can impact study and work. Long-term insomnia results in problems such as daytime dizziness, poor appetite, dyspepsia, and headache. At present, the drugs used for insomnia can be roughly divided into non-benzodiazepines, benzodiazepines, melatonin receptor agonists, antidepressants, and orexin receptor antagonists, all of which are prone to drug tolerance and addiction (1). This deficiency of drug treatment leads people to look for newer and better choices. To reduce the adverse reactions of patients with insomnia after treatment, research has focused on natural drugs and nutrients. Some studies have shown that the intake of certain nutrients and dietary supplements can improve sleep, in terms of both the length and quality (2). Compared to drugs, foods are more suitable for treating sleep diseases because they are non-toxic, have no withdrawal symptoms, and have higher efficacy (3). Among natural drugs, valerian, *Hypericum perforatum*, passionflower, and hops are all considered sleeping herbs for patients with insomnia (4). Valerian, forsythia perforatum, passionflower, and hops are all considered sleep aids for insomnia patients in natural medicines, and valerian is often used in western countries to help sleep or for sedation. As early as the sixteenth century, Valerian Sencha was used in Europe to help sleep and prolong life, and the U.S. Food and Drug Administration has listed valerian as a food supplement, and its use is not contraindicated (5). Its volatile components are often considered to be its effect on insomnia. However, there are also some scholars who debate the effectiveness of valerian in treating insomnia (6–8). This may because of the lack of clarity regarding its composition and dosage in the treatment of insomnia. Therefore, it is very important to clearly establish the dosage and composition of valerian for the treatment of insomnia, and to explore the mechanism by which valerian alleviates insomnia. At present, the mechanism of action of treating insomnia mainly focuses on the following aspects: hypothalamic pituitary adrenal axis (HPA axis) (9), changes in vagus nerve tension, decline in the function of melatonin (MT) system, the influence of inflammatory response factors, disorder of central neurotransmitters, and abnormal function or structure of the limbic cortical system loop (10–12).

A transcriptome is a collection of all transcripts produced by a species or specific cell type. Transcriptome research can facilitate the study of gene function and structure at the overall level, and reveal the molecular mechanism of specific biological processes and disease occurrence. Consequently, transcriptome research has been widely used in basic research, clinical diagnosis, drug R&D, and other fields. Transcriptome analysis based on high-throughput sequencing provides a method to quickly identify the changes in mRNA after drug treatment, which is helpful to understand and explain the mechanism of action. However, to our knowledge, no scholars have studied the transcriptome sequencing of valerian essential oil for treating PCPA model insomnia rats. The transcriptome provides important information about gene structure, expression, and regulation (13). Therefore, we used transcriptome sequencing technology to establish the mechanism by which valerian essential oil alleviates insomnia.

Network pharmacology, which integrates the ideas of system biology and multidirectional pharmacology, has rapidly become the research strategy of new drug discovery and creation. Traditional network pharmacology often ignores the influence of the content of active ingredient on drug efficacy. Therefore, oral bioavailability (OB) and drug composition are two important parameters to weight, so as to reduce the influence of component content difference on drug efficacy. We comprehensively consider oral bioavailability and compound component content (14) to rank the results of Gene Ontology (GO) and Kyoto Encyclopedia of Genes and Genomes (KEGG) pathway analyses according to the change in ranking. We will explore targets and pathways related to components and diseases.

In the current study, we evaluate the key targets and pathways of valerian volatile oil through weight coefficient network pharmacology, identify the molecular mechanism of insomnia based on transcriptome sequencing of an insomnia rat model, study the effect of oral valerian essential oil on brain sedation and hypnosis in PCPA-induced insomnia rats, and discuss its underlying mechanism, with the aim to provide a theoretical basis for further R&D of valerian volatile oil in hypnotic products.

## Materials and methods

### Gas chromatography mass spectrometer component identification

The volatile oil of valerian (Purchased from Guizhou Miao Pharmaceutical Biotechnology Co., Ltd) was identified using GC-MS (Agilent Technologies Inc, 7890 GC / 5977 MS) analysis. The chromatographic conditions were as follows: HP-5 MS capillary column (30 m × 250 μm × 0.25 μm) with a programmed ramp-up mode, starting at 60°C, ramping up to 110°C, at 2°C/min; ramping up to 140°C, at 1°C/min; ramping up to 250°C, at 2°C/min; without splitting, with helium as a carrier gas, with a flow rate of 1 mL/min, and an inlet temperature of 250°C. The mass spectrometry conditions were as follows: signal acquisition in full scan mode; ionization source, EI; ionization energy, 70 eV; transmission line temperature, 280°C; ion source temperature, 230°C; quadrupole temperature, 150°C; solvent delay time, 3 min; and scan mass range, 20–450 amu (15, 16).

### Characterization of the volatile oil components of valerian

The valerian volatile oil data were processed using data analysis software, searched using the NIST standard spectral database. The components were screened based on the match, retention index, and relevant literature. The retention indices of the components in valerian volatile oil were calculated based on the retention indices of n-alkanes (17) with the following equation.


(1) $$\text{RI}=100\text{Z}+\frac{100[\text{tR}\left(\text{X}\right)-\text{tR}(\text{Z})}{\text{tR}\left(\text{Z}+1\right)-\text{tR}\left(\text{Z}\right)}$$

In the formula: tR is the retention time, X is the compound to be analyzed, Z and Z + 1 are the number of carbon atoms of the two n-alkanes before and after the analyte, that is, tR (z) < tR (x) < tR (Z + 1).

### Component-target network pharmacological analysis

#### Acquisition of valerian volatile oil constituents and insomnia targets

The Swiss Target Prediction database and Metafisher database were used to predict the targets of volatile oil components of valerian, and to obtain information about the volatile oil components of valerian targets. The GeneCards database and OMIM database were used to search for targets related to insomnia in combination.

#### Transcriptome sequencing

Transcriptome sequencing is generally the involves sequencing of all of the genes that can be expressed in an organism, and the sequences obtained are compared to those known in public databases to find new genes and predict their functions in general (18). In the current study, hippocampal tissues from three rats were randomly selected from the control, blank, and drug administration groups, and the total hippocampal RNA was extracted using TRIzol lysate. The library was then purified and recovered, the sticky ends were repaired, and the library was constructed by PCR amplification. The library construction and sequencing were performed by Gene Denovo Biotechnology Co., Ltd (Guangzhou, China). Differentially expressed genes (DEGs) were defined as genes meeting the conditions of false discovery rate (FDR) <0.05 and log 2 |FC| ≥ 2. Candidate genes were enriched by GO and KEGG pathway analyses according to the results of differential gene detection.

#### Construction of the “drug-target-disease” interaction network

The intersection targets of the active ingredient, ingredient-disease-differential genes were used to construct the “active ingredient-critical target” network, and the network was visualized by Cytoscape 3.7.2 software.

#### Construction and analysis of the protein-protein interaction network

The potential targets of volatile oil active ingredients, insomnia targets, and the differential genes sequenced by the transcriptome were integrated, duplicate values were deleted, and their intersection was taken and imported into the String (https://string-db.org/) database (19, 20). Next, the minimum interaction threshold was set as “highest confidence” (>0.9), the free protein was hidden, the protein interaction map was obtained, and finally, the results were imported into Cytoscape 3.7.1 software to map their interaction network, the result shown in **Figure 3C**.

### Establishment of weighting coefficients

OB is an important index to measure the efficacy of different formulation dosage forms of drugs. Content determination, as a key element in the quality standard of traditional Chinese medicine to control the content of volatile components, is helpful to control the quality of the formulation and ensure its stability. Therefore, we introduced OB and volatile oil component content (i) as two important factors in the calculation of the new formula.


(2) $$\begin{array}{l} A=OB^{*}I \end{array}$$

(3) $$\begin{array}{l} B=\overset{n}{\underset{i=1}{\sum }}A\text{i} \end{array}$$

(4) $$\begin{array}{l} C=\overset{n}{\underset{i=1}{\sum }}B\text{I} \end{array}$$

In the formula: formula (2) A is the fraction calculated for each active ingredient; formula (3) B represents the sum of the fractions of relevant active components contained in each target, and formula (4) C represents the sum of the fractions of targets contained in each pathway.

### Enrichment analysis of key action target genes GO-BP and KEGG pathways

To further elucidate the gene functions and potential signaling pathway targets of valerian volatile oil in insomnia, the key targets in GO-Biological Process (BP) were analyzed using studio profile. The biological processes and pathways behind them were analyzed through KEGG pathway analysis, and the results of the enrichment analysis were re-ranked according to their weight coefficients. The weighting factor for each pathway is the sum of the weighting factors of all targets in that particular pathway.

### Validation of molecular docking

Based on the enriched targets on the screened pathways, the 3D structure pdb structure files of the target proteins of Prostaglandin-endoperoxide synthase 1 (PTGS1), voltage-dependent N-type calcium channel subunit alpha-1B (CACNA1B), Gamma-aminobutyric acid receptor subunit beta-3 (GABRB3), and HT1AR were downloaded from the RSCB PDB, and the 2 D structure sdf format of the corresponding components were downloaded from the PubChem database. The prepared target protein structure files and compound structure files were imported into Discovery studio 4.0, and the LibDock module was used to dock and calculate the binding energy (21).

### Pharmacodynamic experiments

#### Model preparation and grouping

Forty-eight SPF-grade SD male rats, weighing (200 ± 20) g, were purchased from Xi'an Zhanmeng Biotechnology Co., Ltd, animal license: SCXK (Shaanxi) 2018-001, and this study was approved by the Animal Ethics Committee of Shaanxi University of Traditional Chinese Medicine. After 3 days of adaptive feeding, the animals were divided into six groups according to the random number table: the blank control group, model control group, positive drug group, valerian volatile oil low dose group (20 mg/ml), valerian volatile oil medium dose group (50 mg/ml), and valerian volatile oil high dose group (100 mg/ml), with *n* = 8 animals per group. Except for the blank control group, the rats in each group were injected intraperitoneally with PCPA (300 mg/kg) (MACKLIN D831376) once daily for 3 days to establish a rat insomnia model (22–24). From the fourth day onwards, the blank and model groups were treated with 1% Tween 80 aqueous solution by gavage daily, the positive drug group was treated with 1% Tween 80 aqueous solution containing diazepam (0.092 mg/ml) by gavage, and the drug administration group was treated with 1% Tween 80 aqueous solution containing valerian volatile oil (20, 50, 100 mg/ml) by gavage for 6 days (25–27).

#### Sampling method

After the last administration of each group, the rats were anesthetized with an appropriate amount of sodium pentobarbital at 8:00 a.m. on the next day, and blood was taken from the abdominal aorta under anesthesia, placed in Ethylene Diamine Tetraacetie Acid (EDTA) anticoagulation tubes, left for 30 min, and then centrifuged for 10 min. Subsequently, the upper layer of serum was removed for backup, and the hippocampus of the brain was quickly removed on ice, placed in liquid nitrogen and rapidly frozen, and then transferred to a −80°C for storage.

#### Behavioral observation of rats

In this study, the mine field experiment (28) was used to observe the autonomous behavior of rats in a new environment to explore their behavior and tension. The behavioral laboratory was kept quiet and ventilated, the room was darkened, and the surgical light shone from the ceiling reflected to the experimental operation area. The experimental staff were kept away from the open field analysis box (to the extent that the experimental animals in the box were not visible from the operation angle). Before the experiment, the animals were allowed to acclimatize to the measurement room for at least 10 min. The animal behavior analysis system in the open field analysis box from the upper left, from left to right colored numbering for the same 9 small cells (5 for the central area, and the remaining for the peripheral cells). In each experiment, the tail of the rat was pinched from the root 2/3 lightly into cell 5. Subsequently, the rat was monitored to observe its activities within 5 min. Following the test of each rat, feces were removed and the test chamber and the inner wall were cleaned with disinfectant before wiping with 75% alcohol and blowing dry with a fan. Each rat was tested once. At the end of the experimental period, video analysis, and software statistics were performed. The number of stood on its hind legs and crossed the central grid was recorded.

#### Determination of 5-HT, dopamine and GABA in rat serum by ELISA

ELISA was used to detect the levels of 5-HT, DA and GABA in the serum of each group of rats (29). The standard curve was operated and drawn according to the method in the kit instructions. Using the double antibody sandwich method, the cytokine content in the serum of each group was detected by enzyme marker.

#### Real-time PCR

The total RNA was extracted from rat hippocampal tissue, and the reverse transcription system comprised the following: 5 μl 5 × RT Reaction Buffer, 1 μl 25 mM dNTPs, 1 μl 25 U/μl RNase Inhibitor, 1 μl 200 U/μl M-MLV Rtase, 1 μl Oligo (dt), and 4 μl dd H_2_O (DNase-free). The primer sequence is shown in Table 1, the reaction conditions were as follows: 95°C, 10 min (95°C, 15 s; 55°C, 45 s) × 40; 95°C, 15 s; 60°C, 1 min; 95°C, 15 s; 60°C, 15 s. GAPDH was used as an internal reference to normalize thee data, and the relative expression of genes was measured. Three replicate wells were set up for each experiment, and the data were analyzed using the software that came with the instrument: ABI Prism 7300 SDS Software, and the final data were analyzed at 2^(−Δ*ct*Δ*ct*)^ (30).

**Table 1:** Primer sequence of internal reference gene in qRT-PCR.

**Gene**	**Forward primer (5**′**-3**′**)**	**Reverse primer (5**′**-3**′**)**	**Product length/bp**
5-HT1AR	TCGCTCACTTGGCTCATTGG	TGACAGTCTTGCGGATTCGG	220
ADCY5	AGGTGGATGACCGATTTG	AGGGTGCTGTTCTTCTTG	241
PKA	TTGCCAAGCGTGTGAAAGG	TAACCAGCAGCCATCTCGTAG	145
GABRD	AACGCCATTGTCCTCTTCTC	CCTTCTTTGCCTCCACTTCTAC	130
GAPDH	GTCGGTGTGAACGGATTTG	TCCCATTCTCAGCCTTGAC	181

#### Immunohistochemistry

Paraffin sections of rat brain tissue were repaired by antigen, closed by endogenous peroxidase plus primary antibody 5-HT1AR (BIOSS, bs-1124R, 1:800), ADCY5 (BIOSS, bs-3922R, 1:1,000), PKA (CST, #4782, 1:1,000), GABAA (BIOSS, bs-2252R, 1:1,000), and incubated overnight at 4°C. The sections were removed and left at room temperature for 40 min before washing three times with phosphate buffered saline (PBS) for 3 min each time. HRP-labeled secondary antibody (rabbit antibody, Biyuntian, A0208, 1:1,000) was added and incubated at 37°C for 60 min before washing three times with PBS for 3 min each time. Next, the sections were subjected to DAB (Shanghai Long Island Biotechnology Co., Ltd., FL-6001) staining, rinsing with tap water, hematoxylin re-staining, dehydration and transparent, and then sealed and photographed. Three high magnification fields were randomly selected for observation in each section, and the average absorbance values of positive cells were measured using Image Pro-Plus image analysis software (31).

#### Western-blot

Total hippocampal proteins were extracted, and used for electrophoresis. The membranes were routinely transferred and washed with 5% skim milk powder overnight at 4°C. The antibodies were diluted according to the instructions, before adding to the closure solution and diluted to the desired concentration. The membranes were incubated at room temperature for 2 h. The membranes were incubated with primary antibodies 5-HT1AR (BIOSS, bs-1124R, 1:150), ADCY5 (BIOSS, bs-3922R, 1:100), PKA (CST, #4782, 1:100), and GABAA (BIOSS, bs-2252R, 1:100) were washed thrice with tris buffered saline+Tween (TBST) for 15 min each time. Subsequently, according to the dosage, the membranes were washed with a 1:1,000 dilution of HRP-labeled secondary antibody(Biyuntian, A0208) and incubated with the membranes at 37°C for 1 h. The membranes were washed three times with TBST for 15 min each time and imaged dropwise with Electrochemiluminescence (ECL) luminescent solution (22).

### Statistical analysis

All of the experimental data were statistically analyzed using SPSS 19.0 software. Results are expressed as means ± standard deviations (SDs). One-way analysis of variance (ANOVA) was applied to determine the statistical significance of the differences between the groups, *P* < 0.05 and *P* < 0.01 were regarded as significantly different.

## Results

### GC-MS substance composition analysis

**Figure 1:**
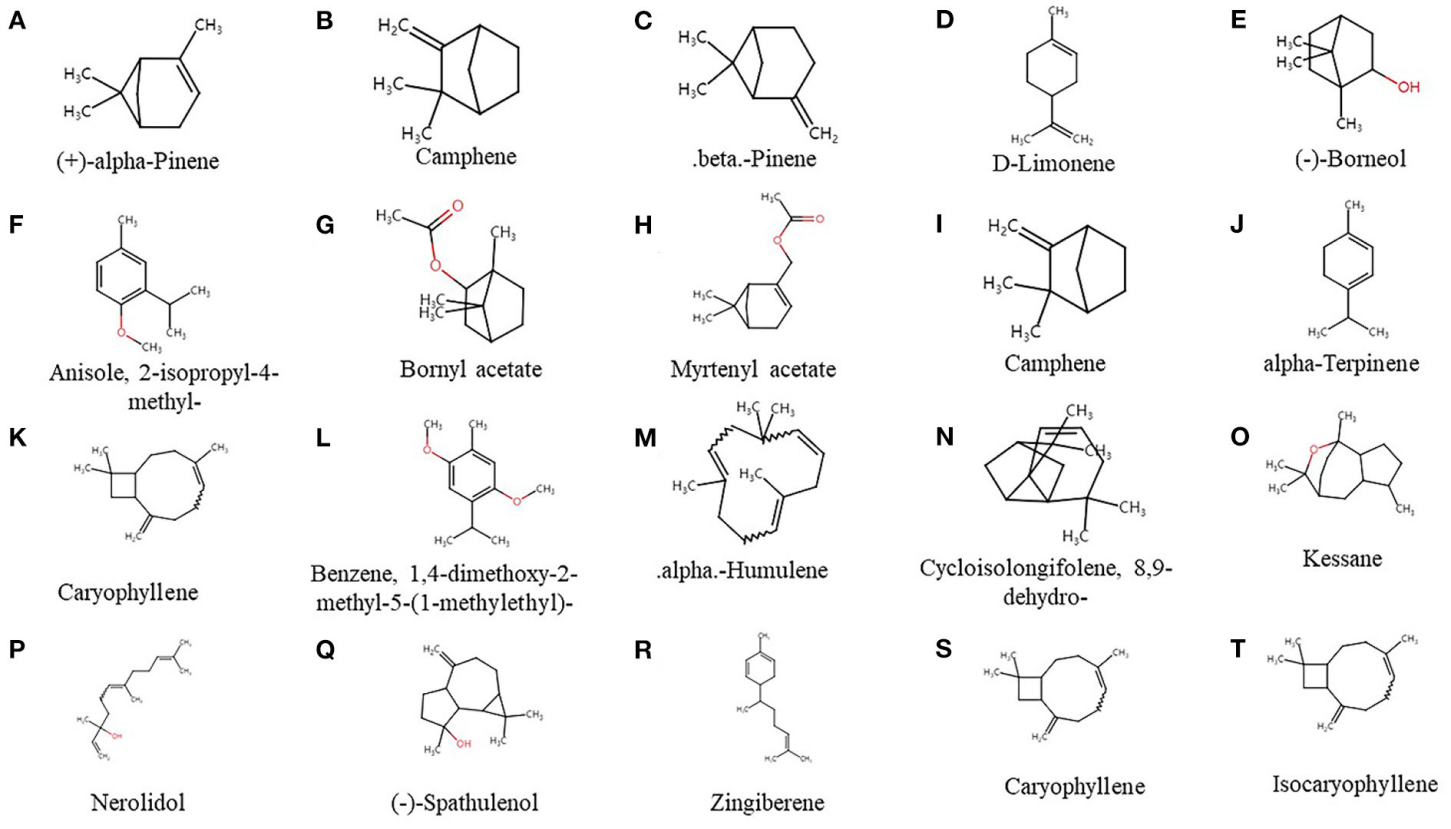
**(A–T)** Chemical structures of 20 active components.

GC-MS was used to analyze the composition of valerian essential oil, and its ion mass spectra were obtained (Figure 3A). Next, the results were searched by the NIST library, and the results were combined with the retention index for characterization. Consequently, 20 active ingredients were obtained; their structural formulae are shown in Figure 1, and the specific composition information is shown in Table 2.

**Table 2:** Qualitative results of valerian essential oil by GC-MS.

**No**.	**Compound**	**CAS**	**RI**	**Area Pct**
1	(+)-Alpha-Pinene	007785-70-8	432.8122969	7.6523
2	Camphene	000079-92-5	450.6271936	22.1145
3	Beta-Pinene	000127-91-3	478.7534122	5.9502
4	D-Limonene	005989-27-5	1,030.389279	3.2338
5	(-)-Borneol	000464-45-9	1,176.573891	1.1534
6	Anisole, 2-isopropyl-4-methyl-	031574-44-4	1,236.436321	0.6647
7	Bornyl acetate	092618-89-8	1,298.235687	30.0004
8	Myrtenyl acetate	001079-01-2	1,330.467341	4.1911
9	Camphene	000079-92-5	1,336.882475	0.2905
10	Alpha-Terpinene	000099-86-5	1,353.062802	1.6241
11	Caryophyllene	000087-44-5	1,417.74108	1.6098
12	Benzene,1,4-dimethoxy-2-methyl-5-(1-methylethyl)-	014753-08-3	1,430.37754	0.3351
13	.alpha.-Humulene	006753-98-6	1,450.548368	0.6178
14	Cycloisolongifolene, 8,9-dehydro-	997214-06-9	1,457.595017	1.9822
15	Kessane	003321-66-2	2,066.472963	0.7821
16	Nerolidol	040716-66-3	260.3438238	0.6701
17	(-)-Spathulenol	077171-55-2	273.2509623	1.3233
18	Zingiberene	000495-60-3	1,619.195355	1.2344
19	Caryophyllene	013877-93-5	1,937.032712	9.5324
20	Isocaryophyllene	000118-65-0	1,800.017044	1.3906

### Transcriptome sequencing results

**Figure 2:**
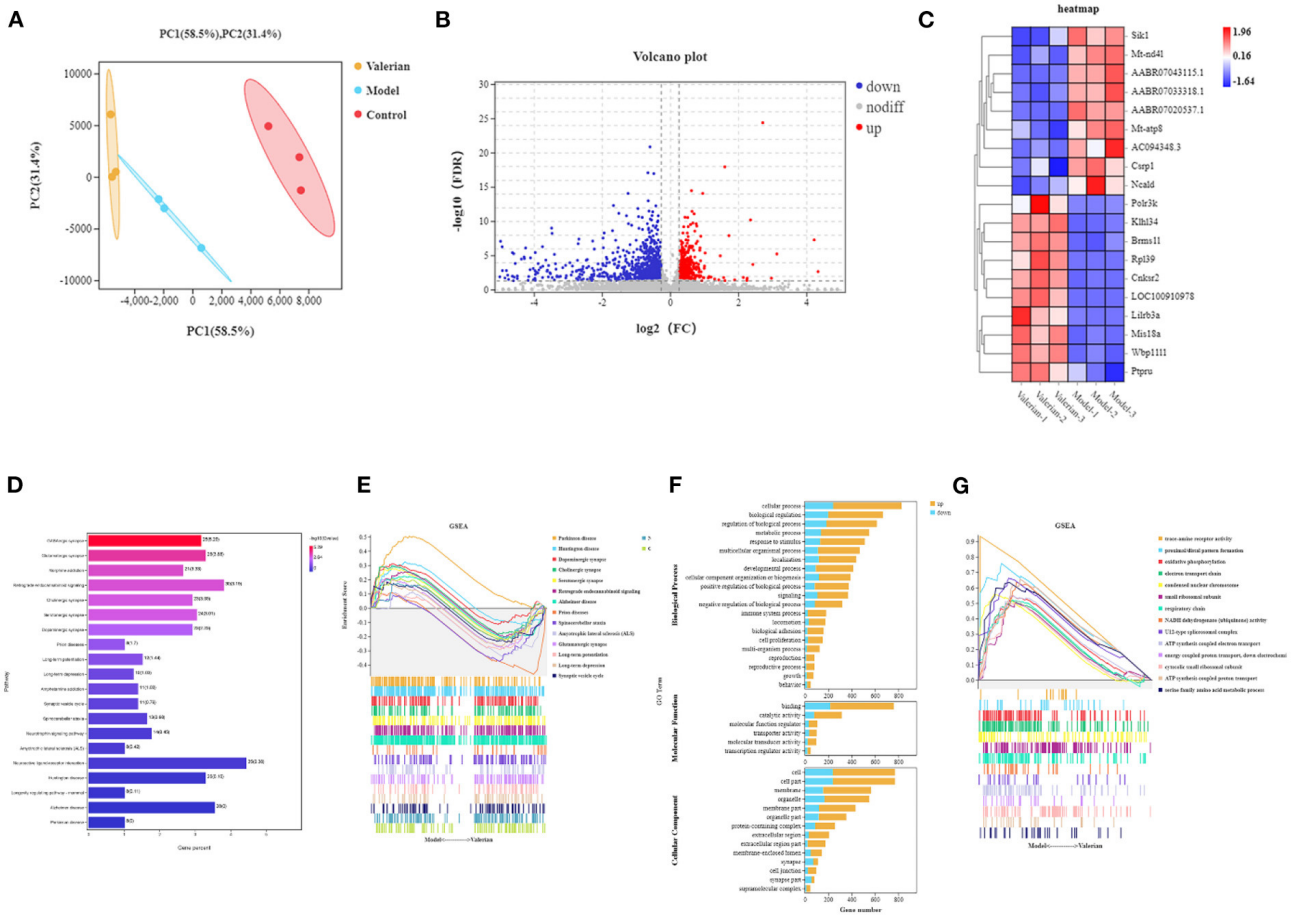
**(A)** PCA cluster analysis diagram. **(B)** Differential gene volcano map. **(C)** Differential gene heat map. **(D)** Enrichment analysis results of sequencing differential gene KEGG. **(E)** GSEA pathway enrichment analysis results. **(F)** Sequencing difference gene go enrichment results. **(G)** GSEA go enrichment analysis results.

Comparing the sequencing results of the blank group, control group and drug administration group, with FDR < 0.05 and |log 2 FC| > 1 as the criteria, 1,558 genes were detected to be significantly differentially expressed, among which 613 genes were upregulated and 945 genes were downregulated. The sample clustering diagram is shown in Figure 2A, the visualization of differential expression is shown in the volcano plot in Figure 2B, and the heat map of differential genes between the control group and drug administration group is shown in Figure 2C.

To investigate the mechanism of action of valerian essential oil for treating insomnia in rats, we performed GO and KEGG pathway enrichment analyses of DEGs. A total of 321 KEGG pathways were enriched for differential genes, among which 20 pathways were related to insomnia, as shown in Figure 2D; these pathways included the GABAergic synapse, glutamatergic synapse, retrograde endocannabinoid signaling, cholinergic synapse, and serotonergic synapse. The differentially expressed genes in the model and valerian oil groups were enriched in 27 biological processes, 12 molecular functions, and 17 cellular components, as shown in Figure 2F. In the valerian essential oil and model groups, the differential genes of molecular functions were mainly enriched in binding and catalytic activity, and the differential genes of cellular components were mainly enriched in the synapse part, membrane-enclosed lumen, and cell part. Differential genes in biological process were mainly enriched in cellular process, metabolic process, response to stimulus, and rhythmic process. Gene Set Enrichment Analysis (GSEA) was used to enrich the obtained differential expression data to obtain closely related genes and signaling pathways; the results showed that they mainly included Parkinson's disease, Huntington's disease, dopaminergic synapse, cholinergic synapse, and serotonergic synapse.

### Construction of the “active ingredient-anti-insomnia targets” network

**Figure 3:**
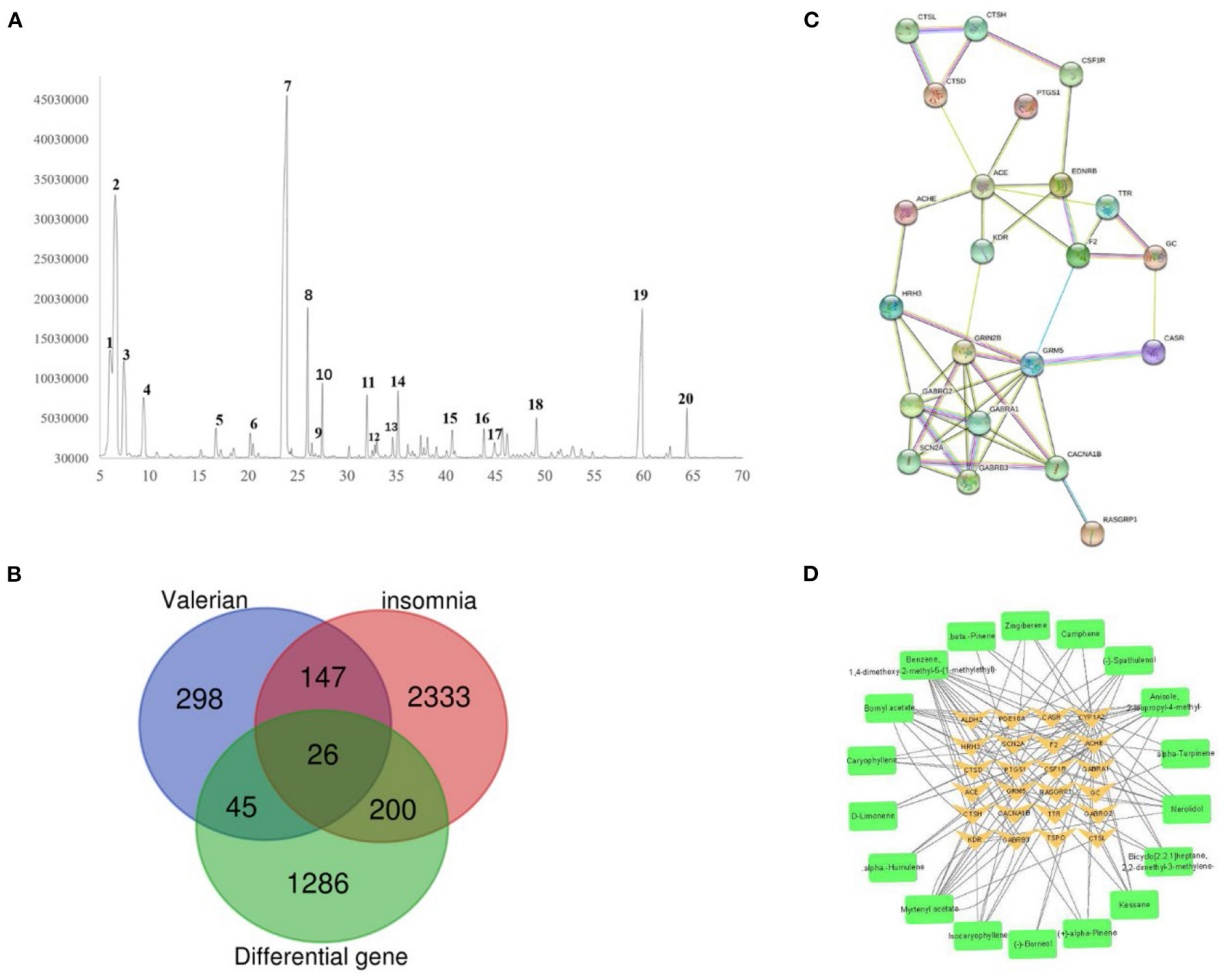
**(A)** Total ion flow diagram of valerian volatile oil. **(B)** Intersection map of valerian essential oil target, insomnia target and differential gene. **(C)** PPI network diagram of key targets. **(D)** Valerian essential oil composition target map.

A total of 516 targets were predicted by Swiss Target Prediction and metafishier; 2,706 insomnia targets and 1,557 differential genes were mined from Genecards, OMIM, and CTD databases; and 26 key genes were obtained by taking the intersection of Draw Venn Diagram. The intersection diagram is shown in Figure 3B, and the screened key targets were imported into Cytoscape 3.7.2 software to build the “active ingredient-key target” network, which is shown in Figure 3D.

### GO and KEGG enrichment analyses based on weighting coefficients

**Figure 4:**
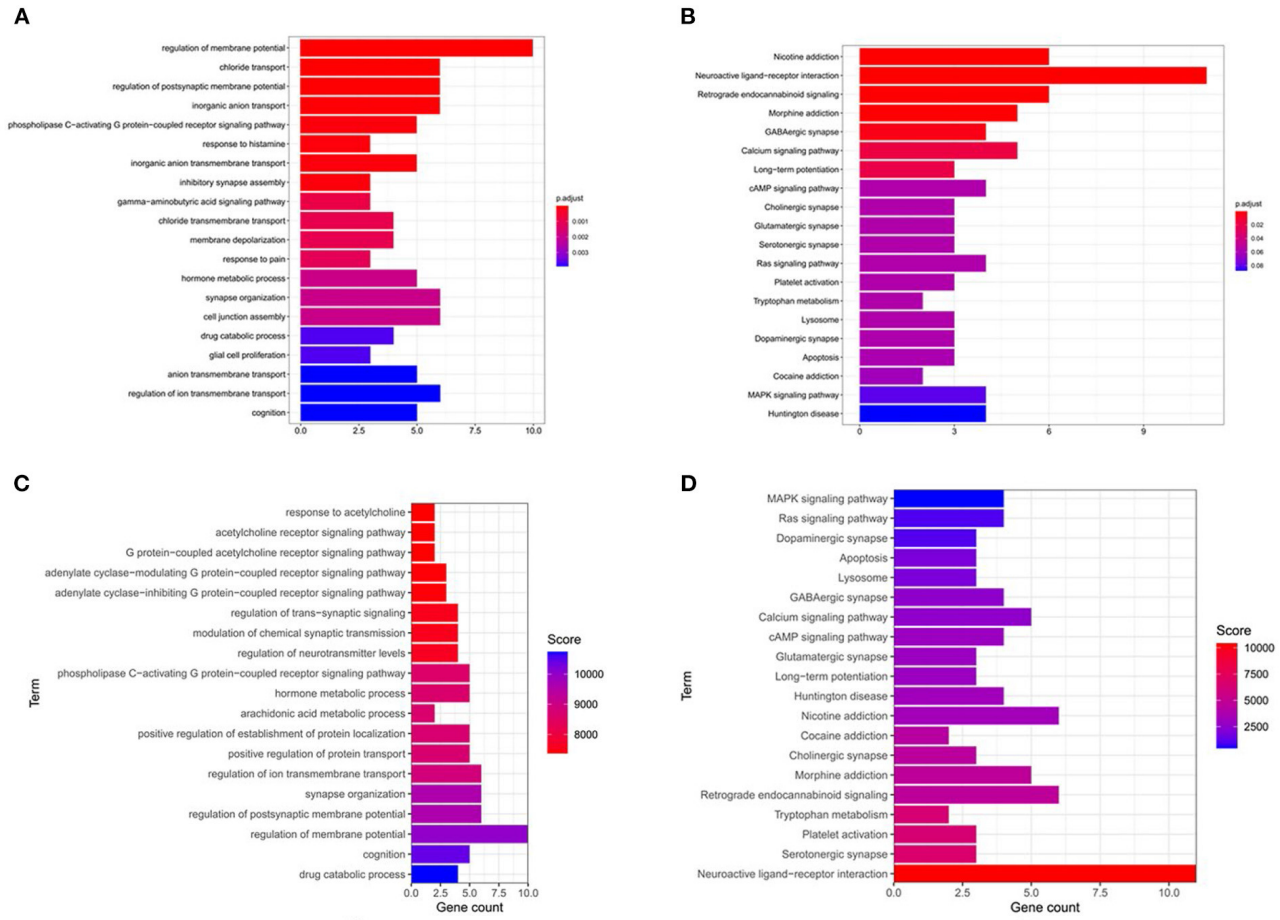
GO-bp and KEGG enrichment results. **(A,B)** The enrichment results of GO-BP and KEGG before sorting. **(C,D)** The GO-BP and KEGG enrichment results after sequencing.

The 26 key targets screened were subjected to GO and KEGG enrichment analyses by R. After the addition of weighting coefficients, the ranking of the biological process (BP) and KEGG pathway enrichment was changed. The top three biological processes were adjusted from the regulation of membrane potential, chloride transport, and regulation of postsynaptic membrane potential to the neuroactive ligand-receptor. The top three KEGG pathways are nicotine addiction, neuroactive ligand-receptor interaction, retrograde endocannabinoid signaling, neuroactive ligand-receptor interaction, serotonergic synapse, Platelet activation. Notably serotonergic synapse rose the 2nd place from the previous 12th place, suggesting that it is very meaningful to study this pathway. Combining the KEGG enrichment analysis (shown in Figure 4) of differentially expressed genes and GESA pathway enrichment analysis (shown in Figures 2E,G), we found that the serotonergic synapse was at the top of the pathways, suggesting that it is an important pathway in the treatment of insomnia by valerian oil.

### Molecular docking validation

**Figure 5:**
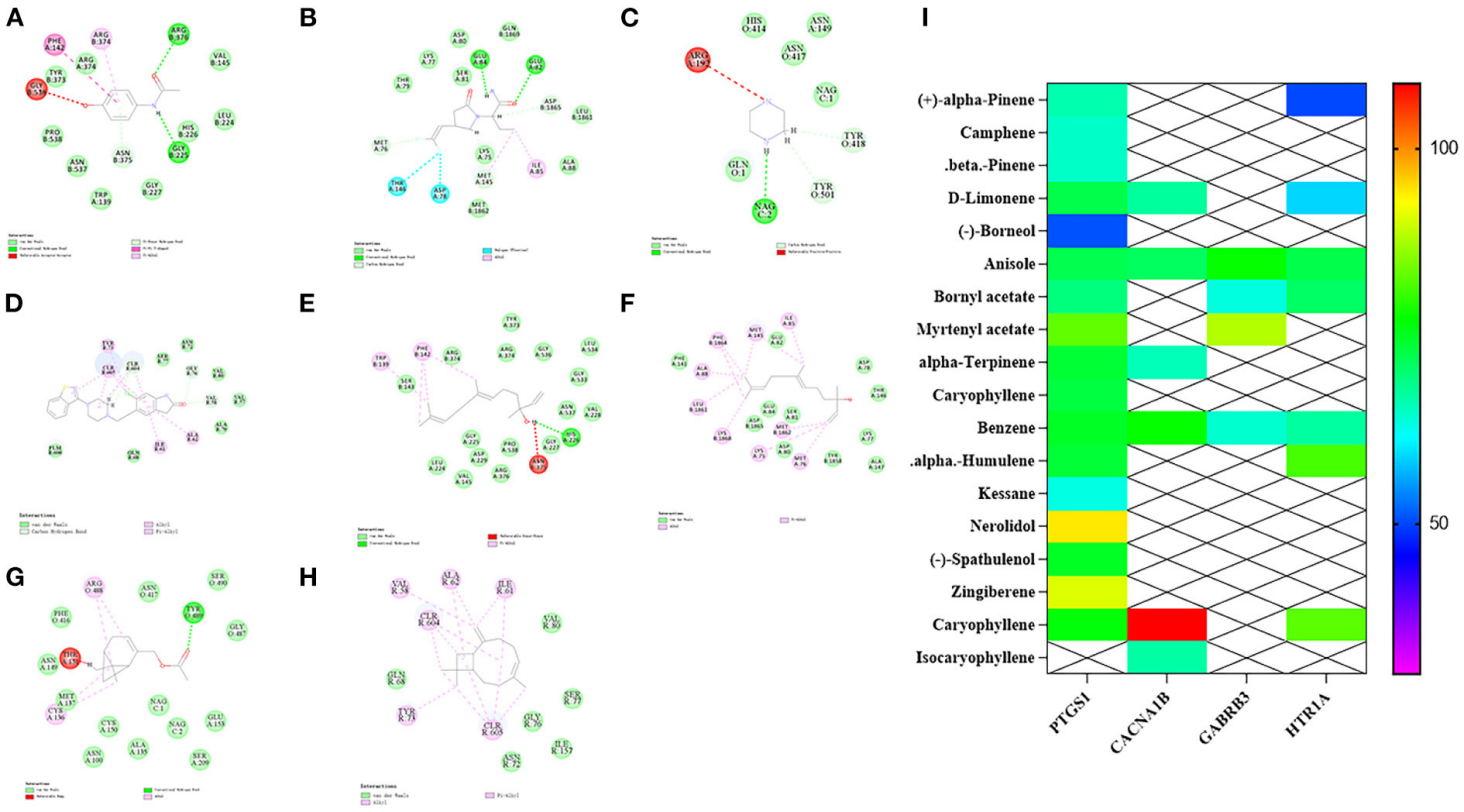
Molecular docking results and heat map display of target and active ingredient. **(A–D)** The docking results of PTGS1, CACNA1B, GABRB3 and HT1AR with positive drugs, respectively. **(E–H)** The docking results of PTGS1, CACNA1B, GABRB3 and HT1AR with nerolidol, caryophyllene, myrtenyl acetate and ziprasidone, respectively. **(I)** Comprehensive heat map display.

According to the three key targets enriched on the serotonergic synapse pathway screened by KEGG (GABRB3, PTGS1, CACNA1B, HT1AR), the ligand molecules were the active components of valerian volatile oil, while acetaminophen, seletracetam, piperazine, and ziprasidone were selected as positive control drugs. Docking was performed using the libdock module in Discovery studio (32), and the degree of docking binding was evaluated using a score. The specific scores of compound and target docking are shown in Table 3. The molecular docking results shown in Figure 5 demonstrate that PTGS1 with the nerolidol component, CACNA1B with the caryophyllene component, GABRB3 with the myrtenyl acetate component, and the scores of HT1AR with caryophyllene and myrtenyl acetate may be the key active components of valerian essential oil for treating insomnia.

**Table 3:** Results of target ligand binding fraction.

Target	Ligand	Score	Target	Ligand	Score
PTGS1	(+)-alpha-Pinene	66.4543	CACNA1B	Anisole, 2-isopropyl-4-methyl-	71.3113
PTGS1	Camphene	64.8275	CACNA1B	alpha-Terpinene	65.821
PTGS1	.beta.-Pinene	64.9379	CACNA1B	Benzene, 1,4-dimethoxy-2-	77.3947
PTGS1	D-Limonene	72.5612	CACNA1B	Caryophyllene	108.663
PTGS1	(-)-Borneol	50.7757	CACNA1B	Isocaryophyllene	66.9982
PTGS1	Anisole, 2-isopropyl-4-methyl-	72.2537	GABRB3	Anisole, 2-isopropyl-4-methyl-	77.3593
PTGS1	Bornyl acetate	69.4778	GABRB3	Bornyl acetate	63.4013
PTGS1	Myrtenyl acetate	83.0315	GABRB3	Myrtenyl acetate	88.223
PTGS1	alpha-Terpinene	73.9346	GABRB3	Benzene, 1,4-dimethoxy-2-methyl-5-(1-methylethyl)-	64.3784
PTGS1	Benzene, 1,4-dimethoxy-2-methyl-5-(1-methylethyl)-	74.8972	GABRB3	Piperazine	43.915
PTGS1	Caryophyllene	73.1767	CACNA1B	D-Limonene	67.5621
PTGS1	.alpha.-Humulene	73.8058	CACNA1B	Anisole, 2-isopropyl-4-methyl-	71.3113
PTGS1	Kessane	63.1315	HT1AR	(+)-alpha-Pinene	50.1324
PTGS1	Nerolidol	94.339	HT1AR	D-Limonene	58.9305
PTGS1	(-)-Spathulenol	74.9981	HT1AR	Anisole, 2-isopropyl-4-methyl-	72.5381
PTGS1	Zingiberene	90.818	HT1AR	Bornyl acetate	71.0268
PTGS1	Caryophyllene	76.6018	HT1AR	Benzene, 1,4-dimethoxy-2-methyl-5-(1-methylethyl)-	67.3647
PTGS1	Acetaminophen	64.2703	HT1AR	.alpha.-Humulene	81.5151
CACNA1B	D-Limonene	67.5621	HT1AR	Caryophyllene	82.6979
CACNA1B	Seletracetam	98.2796	HT1AR	Ziprasidone	87.7035

### General state of rats in each group

**Figure 6:**
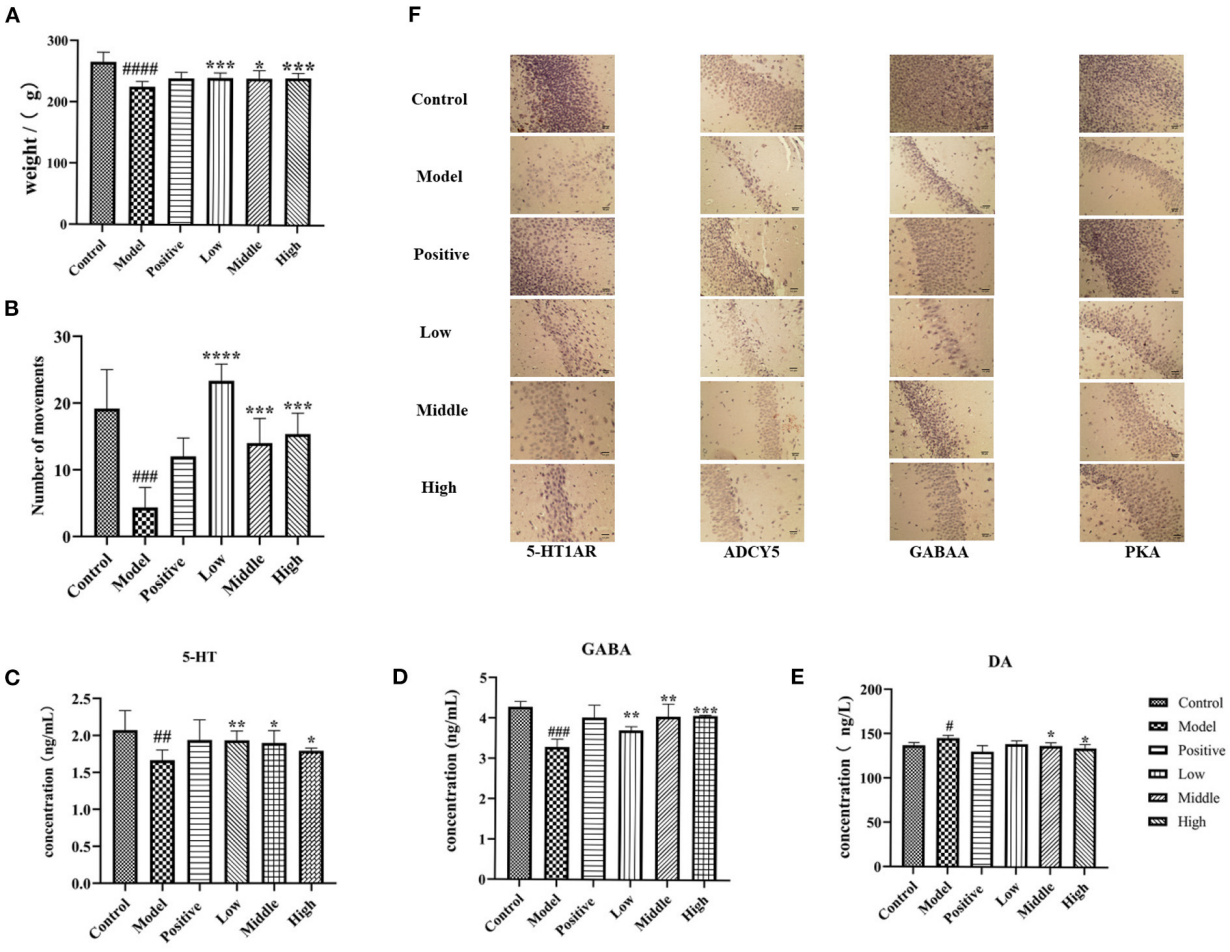
Behavior, contents of GABA, 5-HT and DA in serum and immunohistochemical results of rats in each group. **(A)** The weight changes of rats in each group. **(B)** The behavior times of rats in each group. **(C–E)** The contents of serum GABA, 5-HT and DA in each group. **(F)** The results of immunohistochemistry (compared with the normal group ^#^*p* < '0.05, ^*##*^*p* < 0.01,'^*###*^*p*' < 0.001,'compared with the model group, *p < 0.05, **p < 0.01,'***p < 0.001,'****p < 0.0001).

The general state of the animals was observed. The blank control group was in good mental condition, with shiny hair, regular circadian rhythm, and gradually increasing body mass. After the injection of PCPA, the rats presented with disturbed circadian rhythm, increased daytime activity, irritability, and decreased body mass. Compared to the normal control group, the body mass of rats in the modeling group was significantly reduced. Compared with the model control group, after 6 days of treatment with valerian volatile oil (33), the rats had a normal diet, regular circadian rhythm, and increased body mass, as shown in Figures 6A,B. These phenomena showed that valerian volatile oil had a certain effect on improving the insomnia of rats.

### Effects of different doses of valerian essential oil on serum 5-HT, DA (dopamine), and GABA levels in PCPA insomnia rats

Compared to the normal group, the serum 5-HT and GABA content of the model rats were significantly reduced (*P* < 0.05), while the serum DA content was significantly increased. Compared to the model group, the serum levels of 5-HT and GABA were significantly increased (*P* < 0.05), and the DA levels were significantly decreased (*P* < 0.05) in rats with different doses of valerian essential oil (Figures 6C–E).

### Immunohistochemical results

The results of immunohistochemical staining showed that the sections had a yellowish background, the immune positive cells were varying degrees of yellowish-brown and mainly concentrated in the dentate gyrus granule cells and pyramidal cells in the hippocampal region of CA1 and CA3, and the reactive material was brownish-brown. Moreover, the 5-HT1AR positive reactions were present in the cell pulp and dendrites, and the nuclei did not stain. The results showed that compared to the blank group, the hippocampus of rats in the model group showed significantly decreased expression of 5-HT1AR, GABA, ADCY5, and PKA receptors. Moreover, the expression of 5-HT1AR, GABA, ADCY5, and PKA receptors was significantly increased in the hippocampus of rats in different dose groups of valerian volatile oil group compared to the model group, as shown in Figure 6F.

### Analysis of PCR results

**Figure 7:**
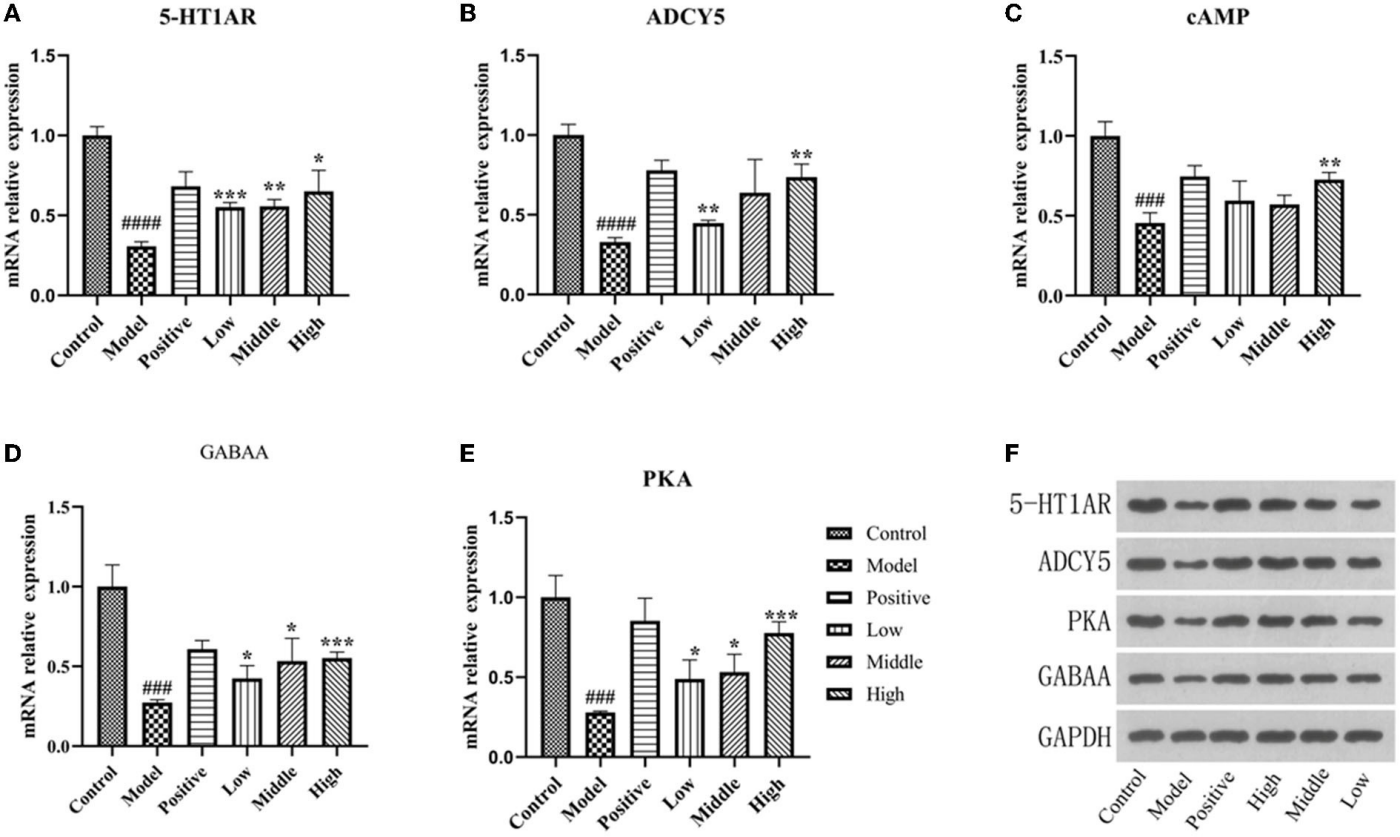
PCR and Western blot results. **(A–E)** The mRNA expression of 5-HT1AR, ADCY5, PKA, and GABAA detected by PCR. **(F)** The protein expression of 5-HT1AR, ADCY5, PKA, and GABAA detected by WB in hippocampus (compared with the normal group ^*###*^*p*' < 0.001,'^*####*^*p*' < 0.0001,'compared with the model group, *p < 0.05, **p < 0.01,'***p < 0.001).

The real-time fluorescence PCR results showed that 5-HT1AR, ADCY5, PKA, GABAA, and cAMP mRNA expression was decreased in the hippocampus of rats in the model group compared to the normal group (*P* < 0.01). Moreover, the mRNA expression of indicator genes was increased in the positive drug group and different doses of the valerian volatile oil group compared to the model group (*P* < 0.01; Figures 7A–E).

### Western blotting analysis

Expression of 5-HT1AR, ADCY5, PKA, and GABAA in the model group was significantly lower than in the normal group (*P* < 0.05), and the protein expression of 5-HT1AR in the group with different doses of valerian essential oil was higher than in the model group, and the results are shown in Figure 7F.

## Discussion

Sleep is a natural state of rest, prevalent such as mammals, birds, and fish, and even in invertebrates such as fruit flies. Characteristics of sleep include reduced active body movements, diminished response to external stimuli, enhanced assimilation (production of cellular structures), and reduced levels of dissimilation (breakdown of cellular structures). In humans, sleep accounts for one-third of life, and it is said that good sleep is the basis for half of the quality of life. Good sleep helps people to recover their strength and brain power and to maintain their health by relieving stress and enhancing memory (34). In contrast, people who do not get enough sleep will experience many problems, such as deficits increative thinking (35), affecting growth and development in adolescents, skin problems, obesity, and other diseases (36). However, the exact mechanism underlying sleep onset has not been fully elucidated. Recent studies on drugs used to treat insomnia drugs have focused on single neurotransmitters; however, a series of neurotransmitters and neuromodulatory substances of many neural networks at different levels of the central nervous system are involved in insomnia, and studied on single neurotransmitters often fail to produce satisfactory results.

Studies have shown that sleep is regulated by the central nervous system and its occurrence is closely related to central neurotransmitters such as 5-HT, GABA, and DA. Glu and GABA play an important role in maintaining the balance of neural excitation and inhibition, and the Glu/GABA ratio is used as an objective indicator to study and evaluate central nervous excitation or inhibition. Decreased GABA levels in the brain increase the Glu/GABA ratio, leading to intense neural activity, such as anxiety and insomnia (37, 38). GABA is mainly distributed in the brain, generated by the decarboxylation of Glu, and is an important inhibitory transmitter that exerts its inhibitory effects mainly by binding to GABAa receptors, causing an inward flow of Cl and inhibiting neuronal firing (39), which is the main neurotransmitter in the activation system, 5-HT was deemed the first neurotransmitter that can play a role in sleep regulation and is recognized as a “sleep-inducing factor,” (40) but its action needs to be mediated by the corresponding receptors. The activity of the 5-HT1AR can induce the production of rapid eye movement sleep and elevate the 5-HT content in the brain to improve the sleep state (41, 42). DA is a wake-promoting substance, mainly acting as an excitatory regulator, and is involved in sleep-wake regulation; DA at normal levels can reduce the number of awakenings and ensure the integrity of sleep (43). Commonly used insomnia models include stress insomnia models, special environment insomnia models, and drug insomnia models. In the early stage of this study, a literature search showed that the serotonin pathway may be the key action pathway of valerian in the treatment of insomnia. So to better explain the specific mechanism, we selected the mouse model of PCPA insomnia and studied the mechanism of action of valerian in the treatment of insomnia. The results of this study showed that the serum 5-HT, GABA decreased, serum DA increased in the chlorophenylalanine (PCPA) model of insomnia, and 5-HT1AR, ADCY5, and PKA in hippocampal tissue. GABAA protein expression content is decreased. It is suggested that valerian essential oil may exert a sedative and hypnotic effect by regulating levels of neurotransmitters such as GABA, DA, and 5-HT.

cAMP, which causes prolonged slow-wave sleep and total sleep time, shortened sleep latency, and has a significant sleep-promoting effect, is involved in sleep and circadian rhythm regulation through PKA. ADCY5 is an adenylyl cyclase that mediates G protein-coupled receptor signaling by synthesizing the second messenger cAMP. Moreover, camp is also a typical second messenger, which responds to the binding of extracellular signals to cell surface receptors through its concentration changes, regulates the activity of intracellular enzymes and non-enzymatic proteins, and thus performs the function of carrying and amplifying signals in cell signal transduction pathways (44, 45). Activation of cAMP-dependent PKA expression increases phosphorylation levels and regulates the expression of genes such as brain-derived neurotrophic factor (BDNF) to exert biological effects (46). The results showed that cAMP mRNA, PKA mRNA, and protein expression were reduced in rats with insomnia, and that valerian essential oil increased hippocampal cAMP, PKA gene and protein expression.

In this study, based on the “weighting factor” network pharmacology and transcriptome sequencing, we found that the serotonergic synapse is a possible pathway of action of valerian oil for insomnia. To further test our conjecture, we used the PCPA method to create a rat insomnia model, administered different doses of valerian essential oil during the experiment and observed the behavior of the rats. The results showed that the intervention of rats with insomnia using valerian essential oil could increased the protein expression of 5-HT1AR, ADCY5, PKA, and GABAA in the hippocampus, increase the serum levels of 5-HT and GABA, decreased the DA level, and increased the mRNA transcripts of 5-HT1AR, ADCY5, PKA, and GABAA in the hippocampus of rats. Moreover, the rats treated with valerian essential oil gradually regained circadian rhythms, stable emotions, moist fur, and a significant increase in body mass.

To further identify the key active components of valerian volatile oil acting in the serotonergic synapse pathway, we selected four key targets in the pathway, PTGS1, CACNA1B, GABRB3, and HT1AR, as receptors, and the active components corresponding to the targets and positive drugs as ligands, and used molecular docking technique to verify the interaction between the main active components of valerian volatile oil. The results showed that nerolidol, caryophyllene, and myrtenyl acetate were the key components of valerian volatile oil in the treatment of insomnia.

**Figure 8:**
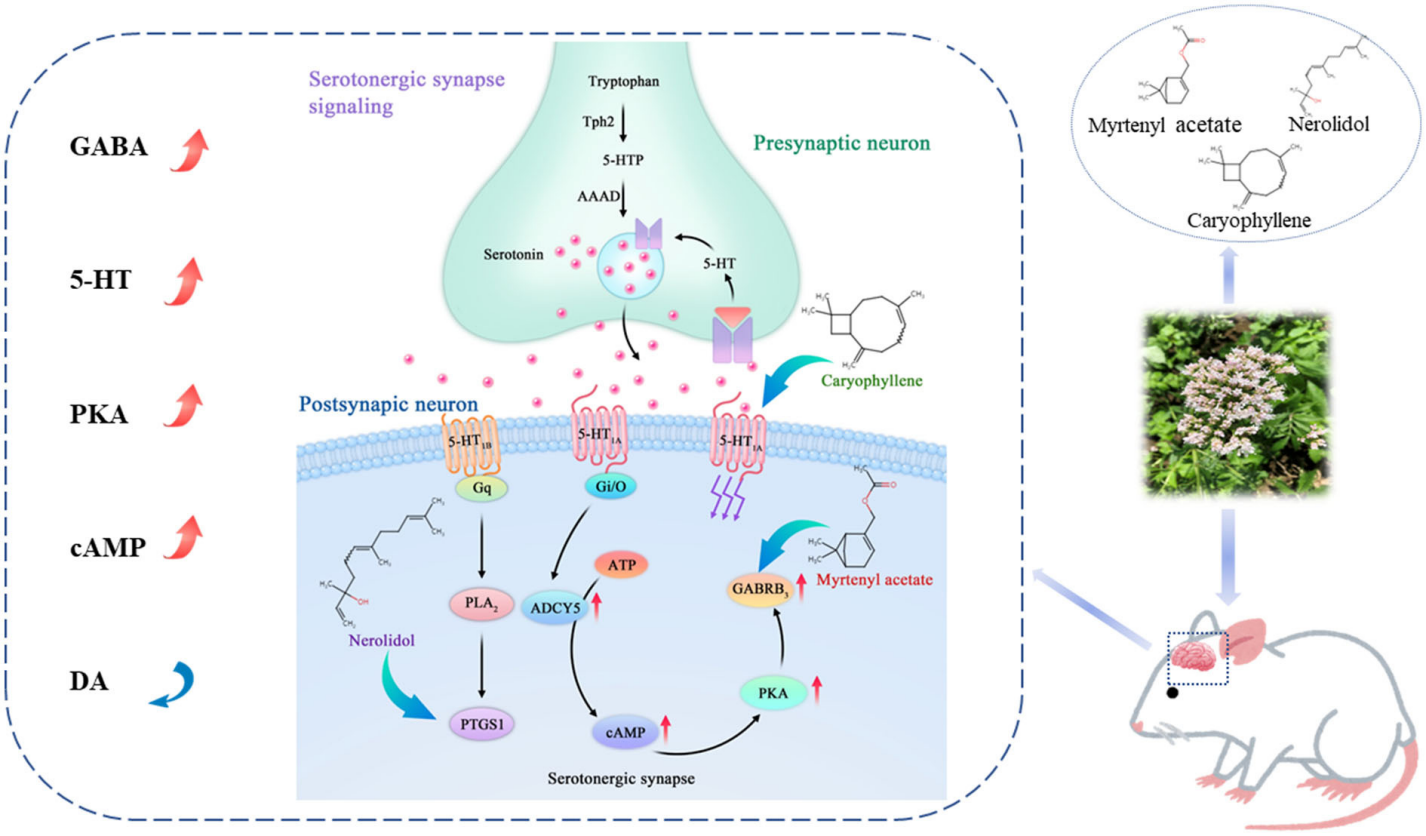
Mechanism diagram.

We integrated transcriptomic techniques, “weighting factor” network pharmacology, and pharmacodynamic experiments to confirm the therapeutic effect of valerian essential oil on insomnia to further elucidate the mechanism underlying its action in the treatment of insomnia. Through further study, we concluded that valerian essential oil acts on the 5-HT1AR receptor through caryophyllene, which upregulates the 5-HT1AR receptor, increases the activity/affinity of the central transmitter 5-HT to increase its release. Subsequently, 5-HT to a G protein-coupled receptor, which is catalyzed by ADCY5 to convert ATP to cAMP and then directly regulate the downstream pathway, activating the core gene protein kinase PKA, which activates the Serotonergic synapse signaling pathway, resulting in increased 5-HT and GABA expression, thus improving insomnia symptoms and relieving anxiety, see Figure 8 for the mechanism diagram. In summary, we believe that valerian, as a medicinal and edible herb, can be considered for development as a health food to alleviate the increasing prevalence of insomnia in humans (46).

This experiment has certain limitations. The causes of insomnia are manifold, but this study only uses PCPA intraperitoneal injection rats to establish insomnia models, so the next step is to use other modeling methods to investigate the efficacy and mechanism underlying the action of valerian essential oil. This experiment only evaluates the pharmacodynamics of rats, and its effect and mechanism in the treatment of insomnia in humans need to be studied further. There are also studies that call into question the safety of valerian after taking it: Sateia showed that valerian can cause mild diarrhea, Leach showed it to be relatively safe. Further clinical studies on its use and safety are needed (47–50).

## Data availability statement

The data presented in the study are deposited in the SRA database repository, accession number PRJNA838430.

## Ethics statement

The study was approved by the Ethical Committee of Shaanxi University of Chinese Medicine.

## Author contributions

WW, XZ, and JS participated in the design of this study and performed the statistical analysis. JZ, YiW, JL, HL, and QG carried out the study and collected important background information. ZW was responsible for the component detection of valerian volatile oil. WW, TL, TT, YuW, and DG drafted the manuscript. YaW, MY, and YS revised the manuscript. All authors read and approved the final manuscript.

## Funding

This project has been supported by Shaanxi Provincial Key Laboratory of Basic and New Drugs of Traditional Chinese Medicine (KF2204), National Natural Science Foundation of China (81703720), Project of Shaanxi Provincial Department of science and technology (2022JM-555), Xianyang Science and Technology Bureau (2021ZDYF-SF-0015), Major Science and Technology R&D Project in Jiangxi Province (20194ABC28009), Shaanxi Provincial Administration of Traditional Chinese Medicine (2021-02-22-014), the 2017 Open Fund of the Key Laboratory of Modern Chinese Medicine Preparation by the Ministry of Education (2017003).

## Conflict of interest

Author ZW was employed by Shaanxi Haitian Pharmaceutical Co., Ltd. The remaining authors declare that the research was conducted in the absence of any commercial or financial relationships that could be construed as a potential conflict of interest.

## Publisher's note

All claims expressed in this article are solely those of the authors and do not necessarily represent those of their affiliated organizations, or those of the publisher, the editors and the reviewers. Any product that may be evaluated in this article, or claim that may be made by its manufacturer, is not guaranteed or endorsed by the publisher.

## Abbreviations

PCPA: P-chlorophenylalanine
SD: Sprague-Dawley
5-HT1AR: 5-hydroxytryptamine receptor 1A
GABA: γ-aminobutyric acid
cAMP: cyclic adenosine monophosphate
PKA: protein kinase A
ELISA: enzyme-linked immunosorbent assay
PCR: Polymerase Chain Reaction
ATP: adenosine triphosphate
HPA: hypothalamic pituitary adrenal axis
MT: melatonin
OB: oral bioavailability
GO: Gene Ontology
KEGG: Kyoto Encyclopedia of Genes and Genomes
GC-MS: gas chromatography-mass spectrometer
DEG: differentially expressed genes
PPI: protein-protein interaction networks
BP: biological process
PTGS1: prostaglandin-endoperoxide synthase 1
CACNA1B: voltage-dependent N-type calcium channel subunit alpha-1B
GABRB3: gamma-aminobutyric acid receptor subunit beta-3
EDTA: ethylene diamine tetraacetie acid
PBS: phosphate buffered saline
TBST: tris buffered saline+Tween
ECL: electrochemiluminescence
DA: dopamine
BDNF: brain-derived neurotrophic factor.
